# The Mitochondrial Genome of the Legume *Vigna radiata* and the Analysis of Recombination across Short Mitochondrial Repeats

**DOI:** 10.1371/journal.pone.0016404

**Published:** 2011-01-20

**Authors:** Andrew J. Alverson, Shi Zhuo, Danny W. Rice, Daniel B. Sloan, Jeffrey D. Palmer

**Affiliations:** 1 Department of Biology, Indiana University, Bloomington, Indiana, United States of America; 2 Department of Biology, University of Virginia, Charlottesville, Virginia, United States of America; Boston University, United States of America

## Abstract

The mitochondrial genomes of seed plants are exceptionally fluid in size, structure, and sequence content, with the accumulation and activity of repetitive sequences underlying much of this variation. We report the first fully sequenced mitochondrial genome of a legume, *Vigna radiata* (mung bean), and show that despite its unexceptional size (401,262 nt), the genome is unusually depauperate in repetitive DNA and "promiscuous" sequences from the chloroplast and nuclear genomes. Although *Vigna* lacks the large, recombinationally active repeats typical of most other seed plants, a PCR survey of its modest repertoire of short (38–297 nt) repeats nevertheless revealed evidence for recombination across all of them. A set of novel control assays showed, however, that these results could instead reflect, in part or entirely, artifacts of PCR-mediated recombination. Consequently, we recommend that other methods, especially high-depth genome sequencing, be used instead of PCR to infer patterns of plant mitochondrial recombination. The average-sized but repeat- and feature-poor mitochondrial genome of *Vigna* makes it ever more difficult to generalize about the factors shaping the size and sequence content of plant mitochondrial genomes.

## Introduction

The mitochondrial genomes of seed plants are exceptionally fluid in size, structure, and sequence complexity, making the adage "no two are alike" applicable in ways that are unparalleled by other organelle genomes. Much of this diversity reflects the accumulation and activity of repetitive sequences. Repeats of diverse size and number have been characterized from the roughly 20 seed plant mitochondrial genomes so far sequenced. At one extreme, the nearly 1 Mb *Cucurbita* mitochondrial genome contains tens of thousands of short (20–40 nt) dispersed repeats that comprise >30% of its genome [1], whereas other genomes contain small numbers of large (1–120 kb) and mostly species-specific segmental duplications [2]. The size and number of repeats in a plant mitochondrial genome is important because they are also the sites of intramolecular recombination, so repeats ultimately underlie much of the known structural diversity in plant mitochondrial genomes as well. Recombination across inverted repeats inverts the intervening sequences, whereas recombination across directly oriented repeats separates the genome into pairs of subgenomic molecules [3], [4]. These processes create a structurally dynamic assemblage of genomic molecules *in vivo* and have led to a virtual scrambling in the gene orders of closely related species [5] and even conspecific genetic lines [2], [6], [7]. Recombination can also cause sequence duplications and deletions, resulting in rapid and sometimes substantial shifts in genome size. For example, although the mitochondrial genomes of five maize cytotypes have virtually identical sequence complexities, a set of large (0.5–120 kb), cytotype-specific duplications has led to >25% variation in genome size [2]. Likewise, a male-sterile strain of *Beta vulgaris* contains an 87 kb duplication that is absent from its fertile counterpart [8], [9]. Recombinationally derived deletions, some of which have important deleterious consequences [10], [11], are common as well.

Recombination frequency is proportional to the size of the repeat: large (>1 kb) repeats recombine at high frequency, intermediate-sized (100–1000 nt) repeats recombine sporadically, and short (<100 nt) repeats are thought to recombine rarely, if ever [7], [12], [13]. Evidence for repeat-mediated recombination traditionally comes from physical mapping of overlapping clones [14], restriction fragment analysis [15], and Southern hybridization studies [4]. More recently, whole-genome sequencing projects based on paired-end sequencing of clone libraries have used conflicting signals in genome assemblies to infer patterns of intramolecular recombination [16]–[18]. Finally, PCR across predicted recombination boundaries has also been used to detect recombinant genotypes [18]. The ability of PCR to amplify low-concentration templates is thought to make it particularly well suited for detection of rare recombinants involving short repeats [7], [19], [20].

In addition to repeat content, seed plant mitochondrial genomes also show substantial variation in gene content, reflecting ongoing gene loss and functional gene transfer to the nucleus [17], [21]. Most gene losses involve ribosomal protein genes and two respiratory genes, *sdh3* and *sdh4* [22], [23]. A survey of some 300 diverse seed plants revealed only two losses of the remaining 24 genes. One of these genes, *cox2*, was found to be universally present across all 300 taxa, save one recent functional transfer to the nucleus in a group of papilionoid legumes [24]–[29]. We sequenced the mitochondrial genome of one of these legumes, *Vigna radiata* (mung bean), confirmed the absence of the *cox2* gene, and discovered a genome in an ongoing state of reduction with respect to gene content. In addition, a comparative analysis of repeat content in the fully sequenced seed plant mitochondrial genomes shows that *Vigna* has a paucity of repeats of all size classes, including the large recombinationally active repeats present in most seed plants. Although PCR revealed evidence of recombinational activity for numerous short repeats, a novel set of control assays showed that methodological artifacts undermine any firm conclusions about the extent of *in vivo* recombination in the *Vigna* mitochondrial genome.

## Results and Discussion

### Genome Assembly and Sequence Content

The *Vigna* mitochondrial genome was sequenced to an average read-depth of roughly 8× following standard protocols for shotgun Sanger sequencing. This included ligation of random 3-kb DNA fragments into plasmid vectors followed by transformation of *E. coli* with the recombinant plasmids. The genome contains one region that is apparently recalcitrant to cloning. A sequence of approximately 100 nt in length, occupying positions 120136–120243 in the genome, was not covered by any of the roughly 2,300 clones generated for the project. PCR and sequencing of this region closed the assembly and revealed two copies of an 11-nt inverted repeat that might have inhibited cloning.

**Figure 1:**
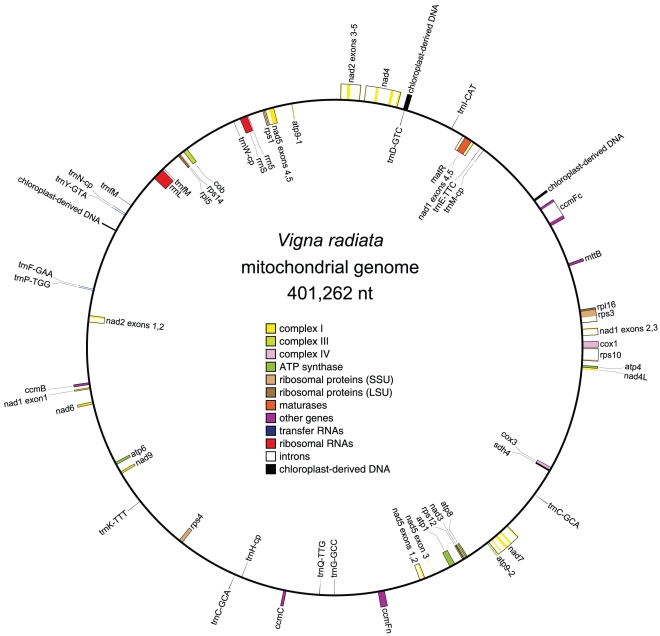
The circular-mapping mitochondrial genome of *Vigna radiata*. Features on transcriptionally clockwise and counter-clockwise strands are drawn on the inside and outside of the circle, respectively.

The *Vigna* mitochondrial genome assembled into a single, circular-mapping molecule of length 401,262 nt and 45.1% GC content, both of which are near the median values of fully sequenced seed plant mitochondrial genomes. The genome contains 31 protein, 3 rRNA, and 16 tRNA genes (Fig. 1). Two identical copies of the *atp9* gene are present in the genome. *Vigna* has one of the most protein-gene-poor mitochondrial genomes so far sequenced in plants, with only two caryophyllids, *Beta* and *Silene*, having fewer intact genes [8], [17]. Like other genome projects (see ref. [30] for discussion), the *Vigna* genome sequence confirms the high accuracy of the inferences of mitochondrial gene content made by Adams et al. [22] in their Southern blot assay of 280 diverse angiosperms. This first completely sequenced legume mitochondrial genome also confirms the absence of the *cox2* gene. The *cox2* gene loss, originally inferred by Southern blot hybridization [28], represents the best-studied case of recent functional transfer of an organellar gene to the nuclear genome, with the transfer restricted to a subset of papilionoid legumes [24]–[29]. Although most other respiratory genes have never been found to have been lost during angiosperm evolution, 17 genes (15 ribosomal protein and 2 respiratory) are known to have been lost frequently [22], [31], [32]. Nine of these 17 genes are either absent from the *Vigna* mitochondrial genome (*rpl2*, *rpl10*, *rps2*, *rps11*, *rps13*, *sdh3*) or are present as pseudogenes in various stages of attrition (*rps7*, *rps19*, *sdh4*). The *sdh4* gene is the most intact of these, with just a single 10-nt insertion located roughly 30 amino acids upstream of the conserved stop codon. Although the insertion drastically alters the downstream reading frame, it does not introduce a premature stop codon, raising the possibility that the *sdh4* gene in *Vigna* is functional, having 1) co-opted a stop codon roughly 15 amino acids downstream of the conserved stop codon, and 2) tolerated substantial 3′ extension and drastic amino acid divergence in the last ∼20% of the conserved length of the gene. Functional studies of the mitochondrial *sdh4* gene, or demonstration of functional transfer of *sdh4* to the nuclear genome, will help resolve these possibilities. Roughly half of the ∼300 nt *rps19* gene is present, albeit in two disparately spaced pieces, whereas just a single 58 nt fragment of the ∼450 nt *rps7* gene remains in the genome. Although sensitive BLAST searches of the genome found a few short DNA fragments (27 and 33 nt in length) with ≥93% similarity to *cox2*, these could easily represent spurious matches. All pseudogene fragments have retained a relatively high (92–98%) sequence similarity to their intact homologs in *Citrullus*, suggesting that pseudogenes are "disappearing" via deletions and/or recurrent reshuffling rather than gradual sequence decay. This stands in sharp contrast to the retention of an essentially full-length *rps14* pseudogene in grasses for some 80 million years [30].

The *Vigna* mitochondrial genome contains a conserved set of 17 *cis*-spliced and five *trans*-spliced group II introns (Fig. 1). Seed plant mitochondrial genomes typically require *trans*-splicing of the intron separating exons 3 and 4 of the *nad5* gene to create a full-length *nad5* transcript. In *Vigna*, exon 3 is identically oriented and less than 3 kb apart from exon 4 (Fig. 1), raising the possibility of a recent reversion to *cis*-splicing of this intron.

As in other seed plants, genes and introns comprise a relatively small fraction, just 16.4%, of the overall *Vigna* mitochondrial genome. BLAST searches revealed only trace amounts of chloroplast- and identifiably nuclear-derived DNA in the intergenic regions, with these two sequence types comprising just 0.5% and 1.6% of the total sequence, respectively (Table 1). These two "promiscuous" sources of DNA typically constitute a more substantial fraction of seed plant mitochondrial genomes [1], [33]. Most nuclear fragments showed similarity to transposable elements, and one fragment matched a lectin protein kinase pseudogene previously found in the mitochondrial genomes of two cucurbits [1]. A large fraction of the non-coding DNA (29.3%, excluding chloroplast- and nuclear-derived sequences) resembles plant mitochondrial DNA from previously sequenced plant mitochondrial genomes or from plant genome projects in the NCBI whole-genome shotgun database (e.g., *Lotus*, *Medicago*, and *Ricinus*), based on a BLAST expect cutoff of 1e-6 (Table 1). One of these regions shows sequence similarity to a group B DNA polymerase and a DNA-directed RNA polymerase, a syntenic arrangement similar to intra- and extrachromosomal plasmids found in other plant mitochondria [34], [35]. The genome also contains two regions with similarity to mitovirus-like RNA polymerases from *Ricinus* and *Vitis* [36].

**Table 1:** Genome coverage by coding and non-coding features in the mitochondrial genome of *Vigna radiata*.

	Feature	# nucleotides	% genome
Coding	Protein exons	28,879	7.2
	*cis*-spliced introns	32,431	8.1
	rRNA	5,258	1.3
	tRNA	1,186	0.3
	Conserved syntenic^1^	84,457	21.0
Non-coding	Mitochondrial-like^2^	117,726	29.3
	Chloroplast-like	2,093	0.5
	Nuclear-like	6,579	1.6
Uncharacterized^3^	—	190,407	47.5

1 Includes all genes, *cis*- and *trans*-spliced introns, and the highly conserved (putatively functional) sequences immediately flanking them

2 Intergenic sequences with similarity to previously sequenced plant mitochondrial DNA, based on a BLAST e-value cutoff of 1e-6 and excluding chloroplast- and nuclear-like sequences

3 The portion of the genome that lacks detectable similarity to sequences in GenBank. The uncharacterized regions, conserved syntenic regions, and identifiable non-coding DNA (mitochondrial-, chloroplast- and nuclear-like) sum to the entire length of the genome

### Repetitive DNA

**Figure 2:**
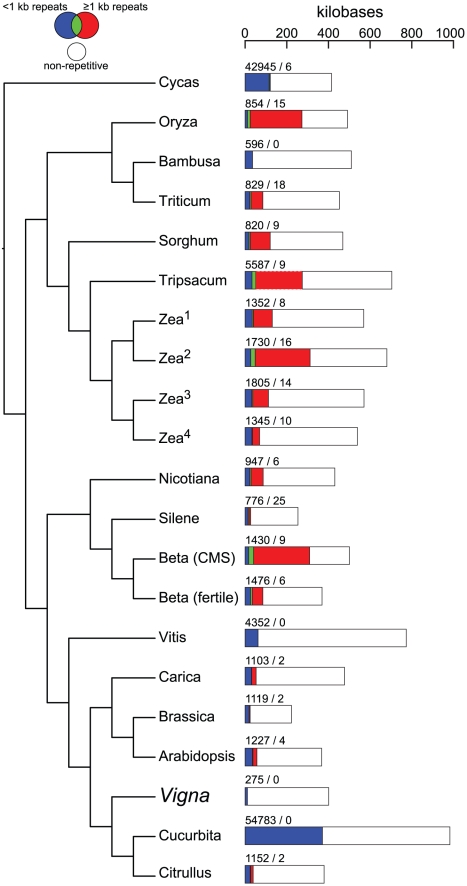
Coverage by repetitive and non-repetitive sequences in fully sequenced seed plant mitochondrial genomes. Genome coverage by repeats <1 kb in length is shown in blue, and coverage by repeats ≥1 kb in length is shown in red. Short repeats are sometimes contained, either partly or entirely, within large repeats; genome coverage by these sites is shown in green. Coverage by non-repetitive portions of the genome is shown in white, so the repetitive and non-repetitive fractions sum to the entire size of the genome. The number of repeats <1 kb and ≥1 kb is indicated directly above each bar. These numbers over-estimate the number of unique repeat coordinates in the genome (see Materials and Methods for details). The four *Zea* genomes are: 1, *Zea mays* subsp. *mays*; 2, *Zea mays* subsp. *parviglumis*; 3, *Zea perennis*; and 4, *Zea luxurians*.

Although the current sample of fully sequenced seed plant mitochondrial genomes is still taxonomically sparse, some preliminary trends in repeat content are emerging. For example, compared to *Cycas* and most eudicots, the nine grass genomes have, on average, a greater proportion of their genomes occupied by large (>1 kb) repeats. Coverage by large repeats varies considerably within grasses and underlies substantial changes in sequence complexity between relatively recently diverged taxa (e.g., *Oryza* and *Bambusa*) as well as subspecies (Fig. 2) and genetic lines [2] of maize. By contrast, eudicot mitochondrial genomes show greater disparities in genome size, but with the exception of the male-sterile genetic line of *Beta*, lower overall coverage by large repeats (Fig. 2). This trend is particularly evident in rosids, in which coverage by large repeats does not exceed 6% for any one species, and in which the two largest sequenced mitochondrial genomes (*Vitis* and *Cucurbita*) contain no large repeats (Fig. 2). Despite these apparent trends, the current sample of genomes is still too sparse, or in some cases too biased (e.g., monocots are represented solely by grasses), to draw firm conclusions about the evolution of repeat content in plant mitochondrial genomes.

**Figure 3:**
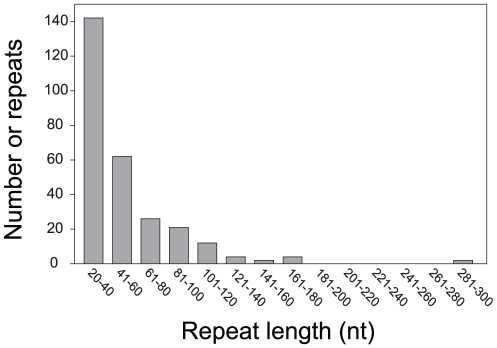
Frequency distribution of repeat lengths in the mitochondrial genome of *Vigna radiata*.

With fewer repeats than all previously sequenced seed plant mitochondrial genomes, *Vigna* represents an extreme with respect to repeat content (Figs. 2 and 3). Repeats contribute very little to the overall size of the *Vigna* genome (just 2.7% coverage compared to 8–62% coverage in other genomes; Fig. 2). The *Vigna* mitochondrial genome is skewed towards fewer and shorter repeats when compared to comparably sized, repeat-poor genomes (e.g., *Bambusa*) or even the much smaller genomes of *Silene* and *Brassica* (Fig. 2). Most *Vigna* repeats are less than 100 nt in length, and most of these are less than 40 nt in length (Fig. 3). The largest repeat in the *Vigna* mitochondrial genome contains a duplicate copy of the *atp9* gene, and at just 297 nt in length, is substantially shorter than the largest repeat in all other fully sequenced seed plant mitochondrial genomes. *Vigna* contains only one copy of the 314-nt recombining repeat that is well-characterized from the mitochondrial genomes of several *Phaseolus* species (a closely related legume) [37]. Finally, *Vigna* is one of a small number of sequenced mitochondrial genomes (including *Bambusa*, *Vitis*, and *Cucurbita*) that lacks the large (>1 kb) recombining repeats that are otherwise characteristic of seed plant mitochondrial genomes (Fig. 3). Mapping studies have shown that *Brassica hirta* lacks large repeats as well [38]. Thus, as they do with genome size [1], mutation rate [39], and RNA editing frequency [40], seed plant mitochondrial genomes also show substantial differences in repeat content and, presumably, recombinational activity.

### Intramolecular Recombination

**Figure 4:**
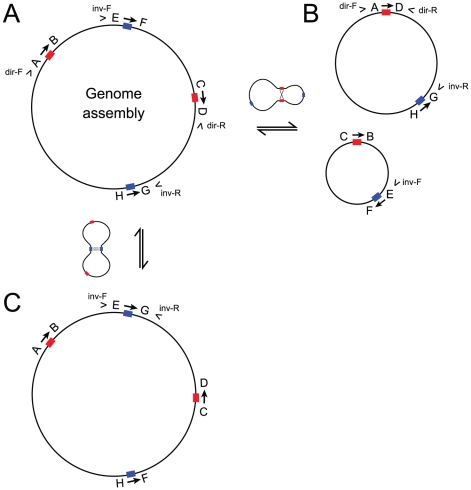
PCR strategy for detecting intramolecular recombination across mitochondrial repeats. Arrows show the orientations of one direct (red) and one inverted (blue) repeat. Arrowheads show the locations and orientations of PCR primers used to detect mitochondrial recombination, relative to the main genome assembly (**A**). Recombination across a direct repeat (red) divides the genome into two circular subgenomic molecules. The altered arrangement of primers dir-F and dir-R permits PCR-based detection of recombinant product A→D (**B**). Recombination across an inverted repeat (blue) inverts the intervening sequences, enabling PCR amplification of recombinant product E→G with primers inv-F and inv-R (**C**).

We detected one chimeric sequencing read that conflicted with the main assembly in that it spanned a predicted recombination boundary involving a 175-nt direct repeat. The discovery of this short and apparently recombinationally active repeat, coupled with the absence of large repeats in the genome, prompted us to screen this and 35 additional short repeats (Dataset S1) for evidence of recombinational activity using the PCR strategy illustrated in Figure 4. Using purified mitochondrial DNA as the template, PCR detected recombinant products for every repeat in our survey, regardless of length (38–297 nt), sequence similarity (93–100%), and orientation (direct or inverted) (Fig. S1). Direct sequencing of PCR products invariably gave results consistent with the expectation for repeat-mediated recombination. The characteristics of six representative repeats from our survey are shown in Table 2, and the corresponding recombinant DNA sequences are available in Dataset S2.

**Table 2:** Characteristics of six representative repeats assayed for recombinational activity in the mitochondrial genome of *Vigna radiata*.

	Repeat		Percent	Copy 1		Copy 2	
Repeat	length	Orientation	identity	Start	End	Start	End
A	175	direct	100	120320	120494	331059	331233
B	104	inverted	98	232468	232571	342673	342776
C	84	direct	100	94981	95064	345737	345820
D	80	inverted	93	148952	149031	161696	161775
E	53	direct	100	335325	335377	392959	393011
F	38	inverted	100	60480	60517	169122	169159

PCR-mediated recombination poses a potential problem when amplifying any kind of repetitive target region (e.g., multigene families and microsatellites) [41]. Although PCR recombination has not, to the best of our knowledge, been reported for the kinds of assays of intramolecular recombination reported here, we wanted to determine whether *in vitro* recombination during PCR could create the patterns observed here and in other PCR-based studies on plant mitochondrial recombination [7], [19], [20]. To do so, we identified four single-copy regions of varying length (55, 90, 148, and 639 nt) and high sequence similarity (94–100%) in the mitochondrial genomes of two different species, *Vigna radiata* and *Cucurbita pepo*, and treated these regions as surrogate repeats in a set of PCR-based recombination assays similar to those described above (Figs. 4 and S2). We used two different PCR templates for these assays. The first was a 1∶1 mixture of total DNA from each species, and the second was an artificial template with substantially higher concentrations of the target regions. We created the latter by separately amplifying the regions of interest from each species then combining the amplicons into a 1∶1 mixture. To test for recombination, we performed PCR using either the total DNA or an artificial amplicon mixture as template, together with primers designed to amplify a bi-species PCR recombination product (Figs. 4 and S2). Because each primer bound to the DNA of a different species, and because our template DNA contained no contiguous and naturally occurring recombinant molecules, PCR-mediated recombination is the only plausible means by which a positive PCR result could be obtained. Using the total DNA mixture as the template, we amplified the intended target region for just two of the eight potential recombination products. These two recombinant products (148 H←F and 639 A→D) were recovered in relatively low yields (Fig. S3). For each of the four bi-species amplicon mixtures, however, we obtained high yields of both possible recombinant products from both dilutions of the artificial template (Fig. S3). Direct sequencing of one high-yield amplicon for each of the eight recombinant products confirmed our prediction of a chimeric, half-*Vigna*/half-*Cucurbita* PCR product.

The higher incidence of PCR recombination in the amplicon templates is consistent with previous findings of increased rates of PCR recombination with increased concentration of template DNA [42], [43]. This is also supported by PCR amplifications of the recombinant configurations from *Vigna* total DNA, as these reactions contained only about 1/70^th^ the level of mitochondrial genomes (see Methods) as the purified mitochondrial DNA template used in the assays described at the beginning of this section. The total-DNA assays gave quite variable results compared to the assays that used purified mitochondrial DNA, yielding (depending on the repeat) either no detectable product, lower levels of product, of comparable levels of product (not shown). Because the total DNA derives from an unidentified and potentially different genetic line than the purified mitochondrial DNA, it is formally possible that mitochondrial repeat content differs somewhat among genetic lines.

These results, together with the bi-species control assays, suggest that many of the *Vigna* recombination products are either present *in vivo* in very low abundance [37] or are actually absent *in vivo*, with their recovery a consequence of PCR-mediated recombination. The bi-species control experiments show that very short regions of sequence identity are sufficient to mediate PCR recombination, the result of either template exchange by *Taq* polymerase [44] or premature extension termination within the repeat and subsequent illegitimate priming by incompletely extended products [41]. Although it is now clear that PCR recombination can mimic patterns of naturally occurring intramolecular recombination in plant mitochondrial genomes, we cannot rule out that at least some, perhaps many, of the *Vigna* repeats actually do recombine *in vivo*, as has been reported for a number of similarly short repeats in the mitochondrial genomes of *Arabidopsis* [7], [19] and *Phaseolus* [20]. The recovery of a recombinant clone involving a short, 175-nt repeat indicates that at least one of the *Vigna* repeats probably does recombine (or has recombined) *in vivo*, but that the recombination products exist at a low enough level that most of them would not be recovered in our relatively low-depth (∼8×) genome assembly. Indeed, quantitative real-time PCR on two recombination products showed that recombinant configurations exist, whether through *in vivo* or *in vitro* recombination, at levels 40–100× less than the main assembly (not shown).

Although Southern blot hybridizations might provide corroborating qualitative and semi-quantitative evidence concerning the recombinational activity of the *Vigna* repeats, Southerns can be insufficiently sensitive for detection of very-low-level recombinant products associated with repeats as short as those in the *Vigna* genome [7], [19], [20], resulting in false-negative evidence concerning recombination. Taken together, the shortcomings of PCR and Southern hybridizations are probably best overcome with whole-genome, paired-end shotgun sequencing. Inexpensive, high-throughput sequencing technologies have the potential to produce deep enough coverage to quantify the relative *in vivo* proportions of dominant and low-level recombinant mitochondrial genome configurations throughout the genome. In the case of *Vigna*, accurate estimation of the relative levels of minor genome configurations will require sequencing the genome to a depth of perhaps 1000–10,000×. Strategies that merge traditional Southern hybridizations with paired-end shotgun data have also proven powerful for understanding the qualitative and quantitative aspects of plant mitochondrial DNA recombination [17]. In the end, high-depth sequencing of the mitochondrial genome of *Vigna*, or any of the growing number of seed plants without large repeats, will ultimately show whether mitochondrial recombinational activity is as notoriously variable across seed plants as are mitochondrial genome size and sequence content [1], mutation rate [39], and RNA editing frequency [40].

## Materials and Methods

### Mitochondrial DNA Isolation, Genome Sequencing and Assembly

Mitochondria were isolated from etiolated seedlings of *Vigna radiata* cv. Berken using the DNAse I procedure [45], and mitochondrial DNA was purified from lysed mitochondria by CsCl centrifugation [46]. A single 3-kb library was constructed, cloned, and Sanger sequenced by the U.S. DOE Joint Genome Institute (JGI) in Walnut Creek, California. Detailed protocols are available at http://www.jgi.doe.gov/sequencing/protocols/prots_production.html. The vast majority of sequence reads were assembled into a single, circular-mapping contig with Phrap (www.phrap.org). Consed was used to visualize and validate the final assembly, and to design PCR primers for filling gaps and augmenting regions of low sequence coverage [47]. The annotated genome sequence is available from GenBank (accession HM367685).

### Genome Annotation

Protein, rRNA, and tRNA genes were annotated as described in Alverson et al. [1]. The mitochondrial genome was also compared to a database of all previously sequenced seed plant mitochondrial genomes with BLAST to identify putatively functional conserved syntenic regions [1]. Briefly, these regions include genes, introns, and the conserved sequences immediately flanking them. The latter are delimited using both syntenic- and sequence-level conservation as determined by BLAST comparison of the *Vigna* genome to a database of all fully sequenced seed plant mitochondrial genomes. These regions are likely to contain promoters, untranslated regions, and *trans*-spliced introns. Chloroplast-derived sequences were identified by comparing the *Vigna* mitochondrial genome to a database of representative seed plant chloroplast genomes with BLASTN, and non-coding mitochondrial-like sequences were identified by searching the *Vigna* genome against a database of all fully sequenced seed plant mitochondrial genomes. All regions that did not match conserved syntenic regions and chloroplast-derived sequences were extracted and searched against the Repbase repetitive element database (ver. 13.05) [48] and the following databases maintained by the National Center for Biotechnology Information (NCBI): the non-redundant (nr) nucleotide and protein databases, the whole genome shotgun (wgs) database, and the est_others database. All NCBI-BLASTN (ver. 2.2.22+) searches used the following settings: word_size 9, gapopen 5, gapextend 2, reward 2, penalty –3, dust no.

### Repeats and Recombination Analyses

Repeated sequences in *Vigna* and other seed plant mitochondrial genomes were identified as described previously [1]. Briefly, the genome was searched against itself using WU-BLAST with the following settings: M = 1, N = 3, Q = 3, and R = 3, kap, span, B = 1×10^9^, and W = 7. All BLAST hits with a BLAST e-value ≤1 were considered repeats. We predicted recombination boundaries for 36 repeats in the *Vigna* genome that varied in length, orientation, and sequence identity, and used Consed [47] to design PCR primers that would amplify one or both predicted recombination products. PCRs were carried out in 25 µL volumes: 18.25 µL water, 2.5 µL 10X buffer (New England Biolabs), 1 µL (400 µM) dNTPs, 0.25 µL *Taq* polymerase (New England Biolabs #M0267L), 1 µL (0.8 µM) per primer, and 1 µL (40 ng) of purified *Vigna* mitochondrial DNA (from cv. Berken) or 2 µL (30 ng) of total *Vigna* DNA (from material of unknown genetic ancestry purchased at local grocery store). Because mitochondrial DNA comprises only about 2% of *Vigna* total DNA [49], the effective concentration of mitochondrial template molecules in PCR carried out using purified mitochondrial DNA was about 70 times that using total DNA. PCR conditions were as follows: 94°C for 3 m, 35 cycles of (94°C for 30 s, 55°C for 30 s, 72°C for 60 s), and final extension at 72°C for 10 m. PCR products were purified using ExoSAP-IT (United States Biochemical, Cleveland, OH), and most were sequenced to verify that we had amplified the expected products. Dataset S1 lists the 36 repeats assayed for recombinational activity in the *Vigna* mitochondrial genome. Recombination primers for six representative repeats (Table 2) are listed in Table S1, and FASTA-formatted sequences of sequenced PCR products are available in Dataset S2.

It is possible that positive PCR results do not reflect the existence of naturally occurring recombinant molecules but instead result from PCR-mediated recombination, which is a concern when amplifying any kind of repetitive target region [41]. To determine whether PCR recombination can give false-positive evidence of intramolecular recombination, we identified identical or near-identical regions shared between the *Vigna* and *Cucurbita* (GenBank GQ856148) mitochondrial genomes (Table S2). As described in Results and Discussion and illustrated in Figure S2, we treated these shared regions as surrogate repeats and performed the same kind of PCR-based assays used to detect recombination in the *Vigna* mitochondrial genome (Fig. 4). PCR conditions were the same as above. The artificial template described in the Results and Discussion was generated by separately amplifying the repeat-containing regions from *Vigna* and *Cucurbita* templates with PCR, gel-extracting the products with a QIAquick Gel Extraction Kit (Qiagen Inc.), then pooling equal volumes of the two PCR products into a single mixture (Fig. S2). Primer sequences for these experiments are listed in Table S3, and FASTA-formatted sequences for sequenced PCR products are available in Dataset S2.

We calculated genomic coverage by repeats and estimated the number of repeats for each of the seed plant mitochondrial genomes shown in Figure 2. Coverage is a non-redundant measure of the number of sites occupied by repeats, as determined by a WU-BLAST of each genome to itself (see above). Short repeats are sometimes contained, either partly or entirely, within larger repeats. When calculating coverage, sites in the genome that fall within two or more such overlapping repeats are counted only once. Repeat number estimates (Fig. 2) are based on the number of unique begin–end coordinates of BLAST hits in the genome. In some cases, this number will over-estimate the actual repeat number, especially for genomes that contain large numbers of imperfect, multi-copy repeat families. For example, *Silene latifolia* contains a family of six recombining direct repeats with a core length of 1362 nt, but with up- and downstream repeat extensions that differ among the six copies [17]. The number of unique begin–end coordinates for a six-copy repeat can range from six (for a six-copy perfect repeat family) to 30 (for a six-copy family of imperfect, variably sized repeats). In this example, WU-BLAST identified 25 different begin–end coordinates for this repeat family (Fig. 2), arguably over-estimating the actual number of repeats by as much as a factor of four.

## Supporting Information

Figure S1 **Short repeats in the *Vigna* mitochondrial genome that showed evidence for recombinational activity.** Repeats vary in length (38–297 nt), sequence similarity (93–100%), and orientation (direct or inverted).
(PDF)

Figure S2 **Outline of an assay to determine whether PCR recombination can mimic plant mitochondrial recombination.** BLAST comparison of the *Vigna* and *Cucurbita* mitochondrial genomes identified surrogate repeats, i.e., regions of identical or near-identical sequence of lengths similar to the repeats in our recombination survey. In all cases, the sequence flanking each side of the "repeat" is unique both within and between the two genomes. Arrows show the orientation of the repeats, and arrowheads mark the location and orientation of PCR primers (**A**). Regions containing the surrogate repeats, shown by gray boxes, were amplified with primer combinations V3+V4 for *Vigna* and C3+C4 for *Cucurbita*, gel-extracted, and the two products were then combined into a 1:1 mixture (**B**). This mixture was used as the template for PCR wherein one primer matched a unique flanking region in *Vigna* and the other matched a unique flanking region in *Cucurbita*. *In vitro* PCR recombination is the only plausible means of obtaining a positive PCR result. Sequencing of this product should reveal a chimeric, half-*Vigna*/half-*Cucurbita* fragment (**C**).
(PDF)

Figure S3 **Results of PCR recombination assays.** Four identical or near-identical regions, each with unique flanking sequences, shared between the *Vigna* and *Cucurbita* mitochondrial genomes served as surrogate repeats for the PCR recombination assays illustrated in Figure S1. The four "repeats" were 55, 90, 148, and 639 nt in length. Lanes are marked as follows: **V**, PCR-amplified "repeat" region from *Vigna*; **C**, PCR-amplified "repeat" region from *Cucurbita*; **V+C/10**, a mixture of the *Vigna* and *Cucurbita* amplicons diluted ten-fold; **T**, amplification of recombination products from a mixture of *Vigna* and *Cucurbita* total DNAs; **A**, amplification of recombination products from undiluted mixture of the **V** and **C** PCR products; **A/10**, amplification of recombination products from a mixture of the **V** and **C** PCR products, diluted 10-fold. We assayed both possible recombination products, which are labeled according to Figure 4.
(TIF)

Table S1 **Primers for PCR assays of intramolecular recombination in the *Vigna* mitochondrial genome.**
(PDF)

Table S2 **Regions of the *Vigna* and *Cucurbita* mitochondrial genomes used for PCR recombination experiments.**
(PDF)

Table S3 **Primers used for PCR recombination experiments.**
(PDF)

Dataset S1 **General Feature Format file with locations, orientations, and percent similarities for 36 repeats assayed for recombinational activity in the *Vigna* mitochondrial genome.**
(TXT)

Dataset S2 **DNA sequences from assays of mitochondrial recombination in *Vigna* and PCR-mediated recombination between *Vigna* and *Cucurbita*.**
(TXT)
