# Supplementary figures and images for: The Mitochondrial Genome of the Legume Vigna radiata and the Analysis of Recombination across Short Mitochondrial Repeats
Source: PLoS One. 2011 Jan 20;6(1):e16404. doi: 10.1371/journal.pone.0016404 (PMC3024419; doi:10.1371/journal.pone.0016404)

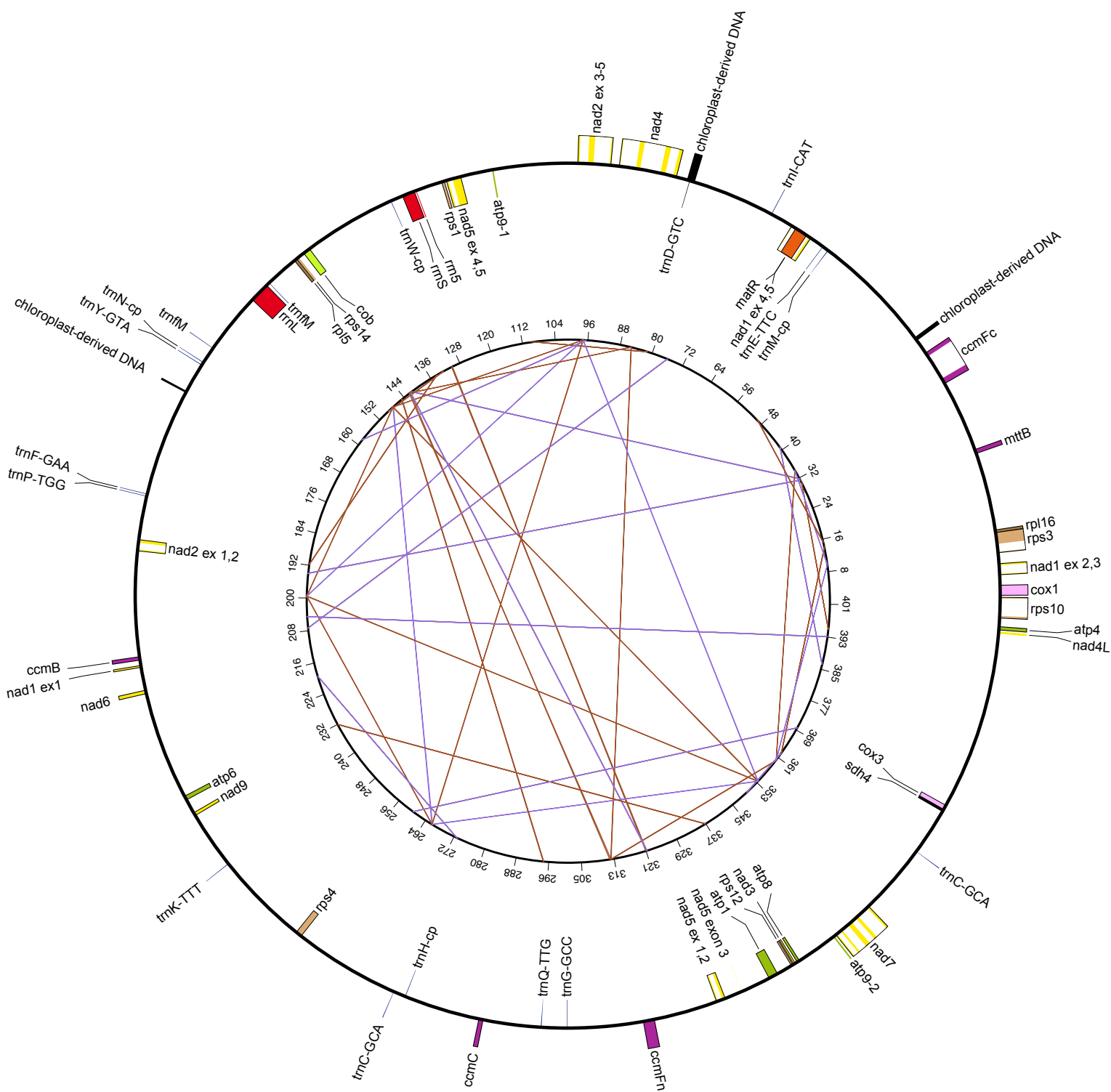

Supplement: Figure S1 — Short repeats in the Vigna mitochondrial genome that showed evidence for recombinational activity. Repeats vary in length (38–297 nt), sequence similarity (93–100%), and orientation (direct or inverted). (PDF) [file pone.0016404.s001.pdf]

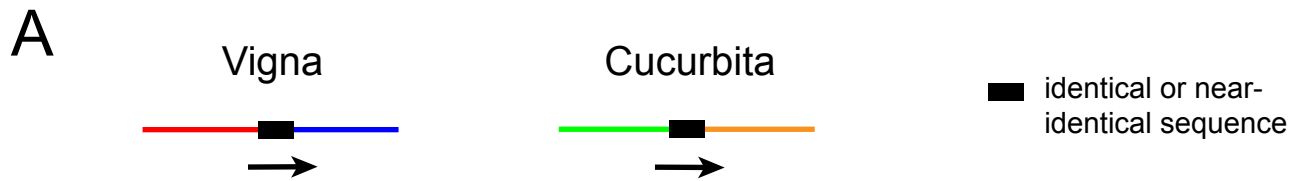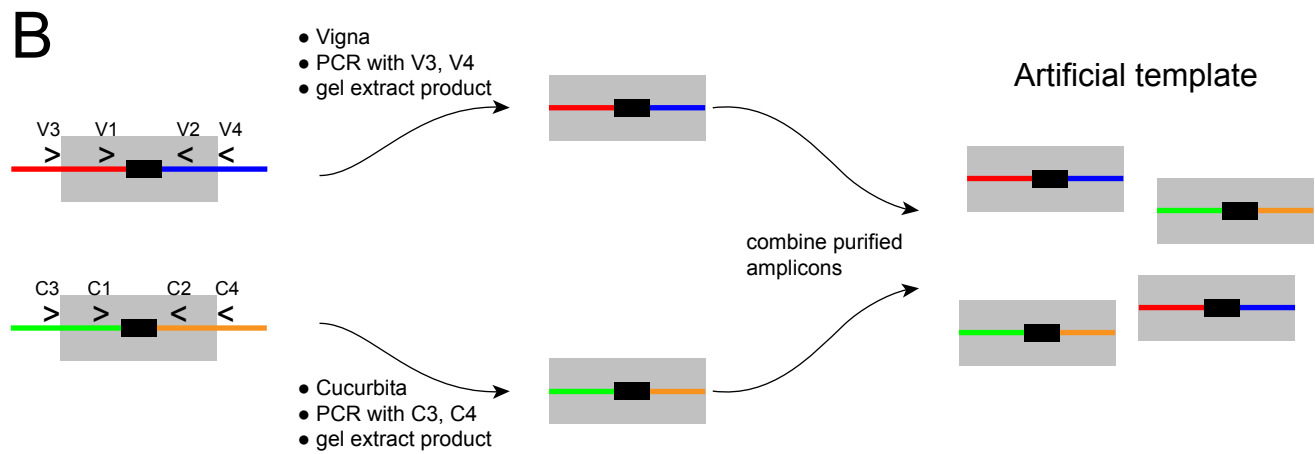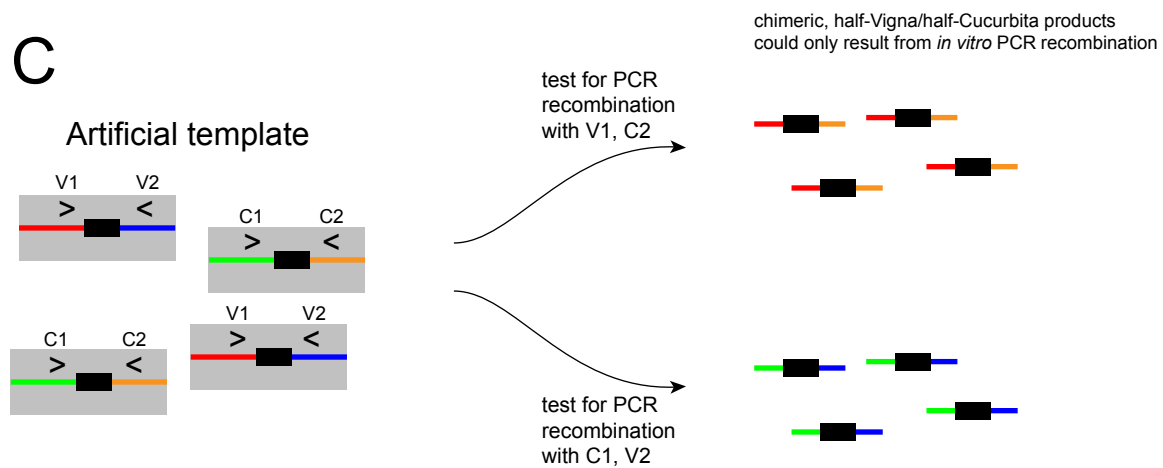

Supplement: Figure S2 — Outline of an assay to determine whether PCR recombination can mimic plant mitochondrial recombination. BLAST comparison of the Vigna and Cucurbita mitochondrial genomes identified surrogate repeats, i.e., regions of identical or near-identical sequence of lengths similar to the repeats in our recombination survey. In all cases, the sequence flanking each side of the "repeat" is unique both within and between the two genomes. Arrows show the orientation of the repeats, and arrowheads mark the location and orientation of PCR primers (A). Regions containing the surrogate repeats, shown by gray boxes, were amplified with primer combinations V3+V4 for Vigna and C3+C4 for Cucurbita, gel-extracted, and the two products were then combined into a 1:1 mixture (B). This mixture was used as the template for PCR wherein one primer matched a unique flanking region in Vigna and the other matched a unique flanking region in Cucurbita. In vitro PCR recombination is the only plausible means of obtaining a positive PCR result. Sequencing of this product should reveal a chimeric, half-Vigna/half-Cucurbita fragment (C). (PDF) [file pone.0016404.s002.pdf]

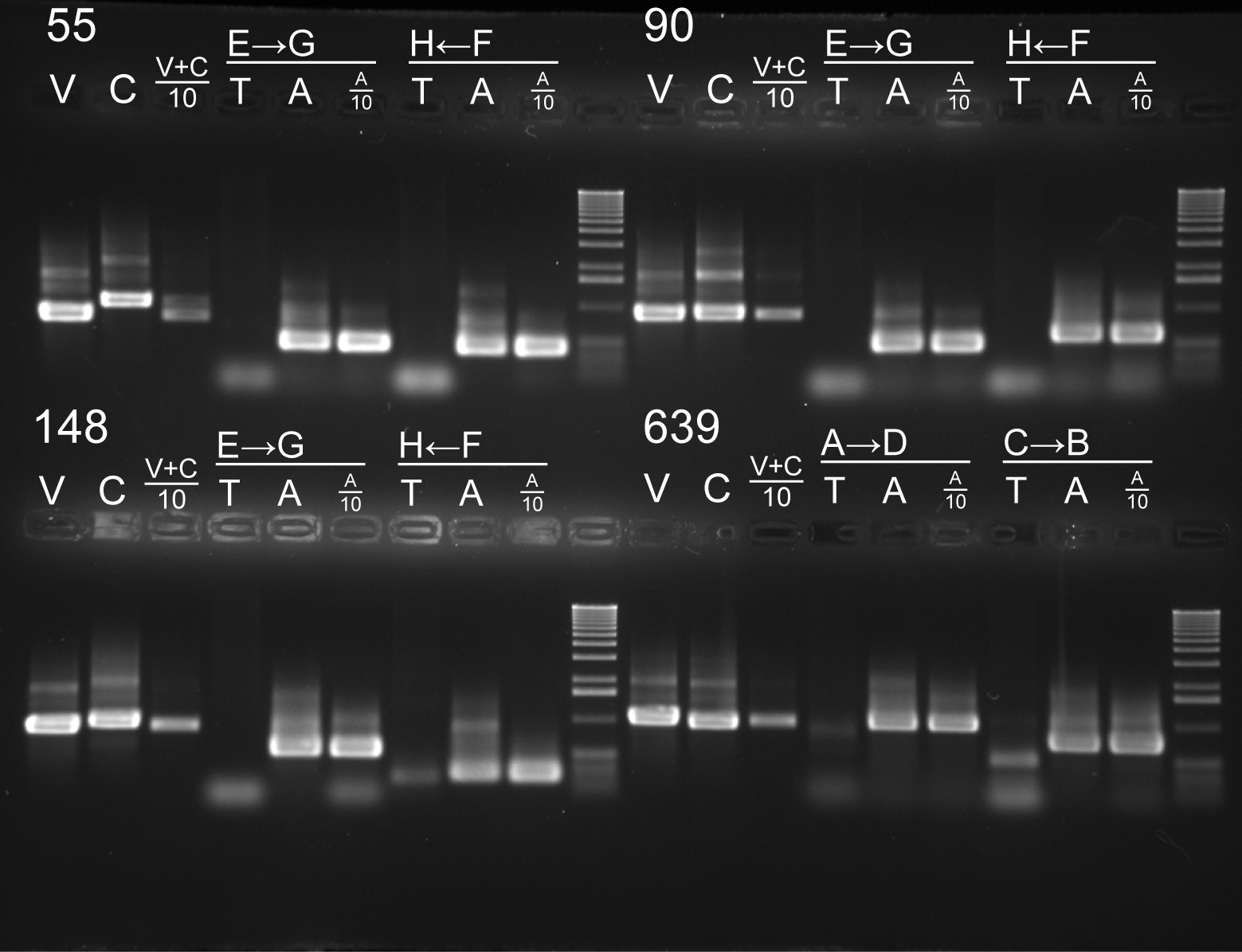

Supplement: Figure S3 — Results of PCR recombination assays. Four identical or near-identical regions, each with unique flanking sequences, shared between the Vigna and Cucurbita mitochondrial genomes served as surrogate repeats for the PCR recombination assays illustrated in Figure S1. The four "repeats" were 55, 90, 148, and 639 nt in length. Lanes are marked as follows: V, PCR-amplified "repeat" region from Vigna; C, PCR-amplified "repeat" region from Cucurbita; V+C/10, a mixture of the Vigna and Cucurbita amplicons diluted ten-fold; T, amplification of recombination products from a mixture of Vigna and Cucurbita total DNAs; A, amplification of recombination products from undiluted mixture of the V and C PCR products; A/10, amplification of recombination products from a mixture of the V and C PCR products, diluted 10-fold. We assayed both possible recombination products, which are labeled according to Figure 4. (TIF) [file pone.0016404.s003.tif]
