# Supplementary material for: The Mitochondrial Genome of the Legume Vigna radiata and the Analysis of Recombination across Short Mitochondrial Repeats
Source: PLoS One. 2011 Jan 20;6(1):e16404. doi: 10.1371/journal.pone.0016404 (PMC3024419; doi:10.1371/journal.pone.0016404)
Supplement: Table S1 — Primers for PCR assays of intramolecular recombination in the Vigna mitochondrial genome. (PDF) [file pone.0016404.s004.pdf]

Table S1a. Primers for PCR assays of intramolecular recombination in the *Vigna* mitochondrial genome. See Table S1b for primer sequences and locations.

Repeat	Forward	Reverse
A	vigna.3	vigna.4
B	vigna.17	vigna.18
C	vigna.27	vigna.28
D	vigna.45	vigna.46
E	vigna.55	vigna.56
F	vigna.69	vigna.70

Table S1b. Primers used for PCR assays of intramolecular recombination in the *Vigna* mitochondrial genome

Primer name	Start coordinate	End coordinate	Strand	Sequence
vigna.3	120142	120162	forward	GAGCATTGATTTGAGTTTTAC
vigna.4	331374	331393	reverse	TCTCTTCTTCCAACCTATTT
vigna.17	232266	232285	forward	ATTTCAATTGATTAACATCG
vigna.18	342123	342141	forward	CCAAACAAAGGGTTCTTTA
vigna.27	94733	94750	forward	GCAAAAGTGGGTTCGTAT
vigna.28	345869	345892	reverse	TAACGTAACAACTAGTATTGAAAG
vigna.38	234996	235016	reverse	AGTAGCAAGAAGGCTTTATTA
vigna.43	209986	210003	forward	GAATGGATCGGTTAAACA
vigna.44	234446	234464	forward	CGGAGATATTCGTGTTACT
vigna.45	148254	148274	forward	TAATATGATACGCTGTGAAGT
vigna.46	161508	161525	forward	TGATGAAACATAATTGCG
vigna.50	210612	210630	reverse	GCATTTATCGCATATTTGA
vigna.55	335125	335147	forward	CTGTAACTTCTCTTTAGCTTATG
vigna.56	393071	393091	reverse	TCTTCGATTACATACCTATCA
vigna.69	60241	60261	forward	CGTACAGCGAGTTATATAGAA
vigna.70	169075	169093	forward	GGCTTGATTTGTTACTTGA
