# Supplementary material for: The Mitochondrial Genome of the Legume Vigna radiata and the Analysis of Recombination across Short Mitochondrial Repeats
Source: PLoS One. 2011 Jan 20;6(1):e16404. doi: 10.1371/journal.pone.0016404 (PMC3024419; doi:10.1371/journal.pone.0016404)
Supplement: Table S2 — Regions of the Vigna and Cucurbita mitochondrial genomes used for PCR recombination experiments. (PDF) [file pone.0016404.s005.pdf]

Table S2. Regions of the *Vigna* and *Cucurbita* mitochondrial genomes used for PCR recombination experiments.

	Vigna		*Cucurbita*			
Region length	Start coordinate	End coordinate	Start coordinate	End coordinate	Relative orientation	Percent identity
55 nt	244766	244820	80801	80855	inverted	100
90 nt	127172	127260	967152	967241	inverted	98
148 nt	123134	123281	882971	883118	inverted	96
639 nt	209620	210258	840038	840676	direct	94
