# Supplementary material for: The Mitochondrial Genome of the Legume Vigna radiata and the Analysis of Recombination across Short Mitochondrial Repeats
Source: PLoS One. 2011 Jan 20;6(1):e16404. doi: 10.1371/journal.pone.0016404 (PMC3024419; doi:10.1371/journal.pone.0016404)
Supplement: Table S3 — Primers used for PCR recombination experiments. (PDF) [file pone.0016404.s006.pdf]

Table S3. Primers used for PCR recombination experiments.

Species	Primer name[1]	Start coordinate	End coordinate	Direction	Sequence
Vigna	55_vigna_recomb+	244484	244505	forward	TTAGCTCTGCAGATGAAGCAAG
	55_vigna_recomb-	245097	245118	reverse	AGAAGATCGAGCTTGTGGAAAG
	55_vigna_templ+	244458	244479	forward	ATTGGCTAGAGCAGTCACTTCC
	55_vigna_templ-	245201	245222	reverse	CCCTCCAAGTTGTTTCAAAGTC
Cucurbita	55_zuc_recomb+	80666	80687	forward	CCTGACCTGCCCTTATTGTTAG
	55_zuc_recomb-	80920	80941	reverse	GAAATTGTTCGTTCACCCTCTC
	55_zuc_templ+	80280	80301	forward	ACCCCCAATCAATAGAACACAC
	55_zuc_templ-	81233	81254	reverse	TCTGACTGTACCGCTTTTCAGA
Vigna	90_vigna_recomb+	126968	126989	forward	CTGGTGTTGGATCACCTACTCA
	90_vigna_recomb-	127505	127526	reverse	GCTTCAAAGATGCTGCTTACCT
	90_vigna_templ+	126852	126873	forward	TTTGGTGGCAGGTCTTTCTATT
	90_vigna_templ-	127727	127748	reverse	AGAATGGCATAGCGATCAAACT
Cucurbita	90_zuc_recomb+	966956	966977	forward	GCGCAGCTTGGTAGTTTAGTTT
	90_zuc_recomb-	967468	967489	reverse	CAATGGAATTGTGTGTGAGACC
	90_zuc_templ+	966709	966730	forward	GAAGAGGGGACTGCTAAATGTG
	90_zuc_templ-	967587	967608	reverse	ATGCAGGCAGTATTCAGAACCT
Vigna	148_vigna_recomb+	122954	122975	forward	TGCTTGGTCTCCCTTTATTTTC
	148_vigna_recomb-	123296	123315	reverse	TCCATTTTCCTTTCCCTTCC
	148_vigna_templ+	122809	122828	forward	GCTTTGTACAGCCCGAGAAC
	148_vigna_templ-	123664	123683	reverse	ACTCGGGAACATGAAGTTGG
Cucurbita	148_zuc_recomb+	882739	882760	forward	AAGCCTACGGTTGCTTAGTCAG
	148_zuc_recomb-	883212	883233	reverse	AATGAGAGAACGGCCAATTAAA
	148_zuc_templ+	882474	882495	forward	AGGGGAAGCACCTACTCCTAAC
	148_zuc_templ-	883385	883406	reverse	AAACTAAGTCGACGCGAAGAAG
Vigna	639_vigna_recomb+	209412	209433	forward	GAGAAATAGATCGGGTTCGTGA
	639_vigna_recomb-	210259	210280	reverse	CAGTTGTCGCTTAAATGCAGAG
	639_vigna_templ+	209334	209355	forward	GTAAGGAGGCCAGACAAGAAAG
	639_vigna_templ-	210357	210378	reverse	TTGTTTGTTGTAAATCGCTGGT
Cucurbita	639_zuc_recomb+	840000	840021	forward	ACCAGCCTTGAAGTGAATGAAT
	639_zuc_recomb-	840786	840807	reverse	TTTCAATAGCTGCCAAAACAGA
	639_zuc_templ+	839968	839989	forward	TTGGTTGAAGATGATGTTACGC
	639_zuc_templ-	840914	840935	reverse	TCAAATAGGTCGGGAAGAAAGA

[1]Primer names with the "recomb" suffix were used to test for recombination, and primer names with the "templ" suffix we[illegible] used to amplify repeat-containing regions, which served as artificial templates for PCR recombination assays
